# Emerging concepts on the mechanical interplay between migrating cells and microenvironment *in vivo*

**DOI:** 10.3389/fcell.2022.961460

**Published:** 2022-09-27

**Authors:** Guilherme Ventura, Jakub Sedzinski

**Affiliations:** The Novo Nordisk Foundation Center for Stem Cell Medicine (reNEW), University of Copenhagen, Copenhagen, Denmark

**Keywords:** *in vivo* cell migration, mechanotransduction, confinement, topography, durotaxis, microenvironment sensing

## Abstract

During embryogenesis, tissues develop into elaborate collectives through a myriad of active mechanisms, with cell migration being one of the most common. As cells migrate, they squeeze through crowded microenvironments to reach the positions where they ultimately execute their function. Much of our knowledge of cell migration has been based on cells’ ability to navigate *in vitro* and how cells respond to the mechanical properties of the extracellular matrix (ECM). These simplified and largely passive surroundings contrast with the complexity of the tissue environments *in vivo*, where different cells and ECM make up the milieu cells migrate in. Due to this complexity, comparatively little is known about how the physical interactions between migrating cells and their tissue environment instruct cell movement *in vivo*. Work in different model organisms has been instrumental in addressing this question. Here, we explore various examples of cell migration *in vivo* and describe how the physical interplay between migrating cells and the neighboring microenvironment controls cell behavior. Understanding this mechanical cooperation *in vivo* will provide key insights into organ development, regeneration, and disease.

## Introduction

**FIGURE 1:**
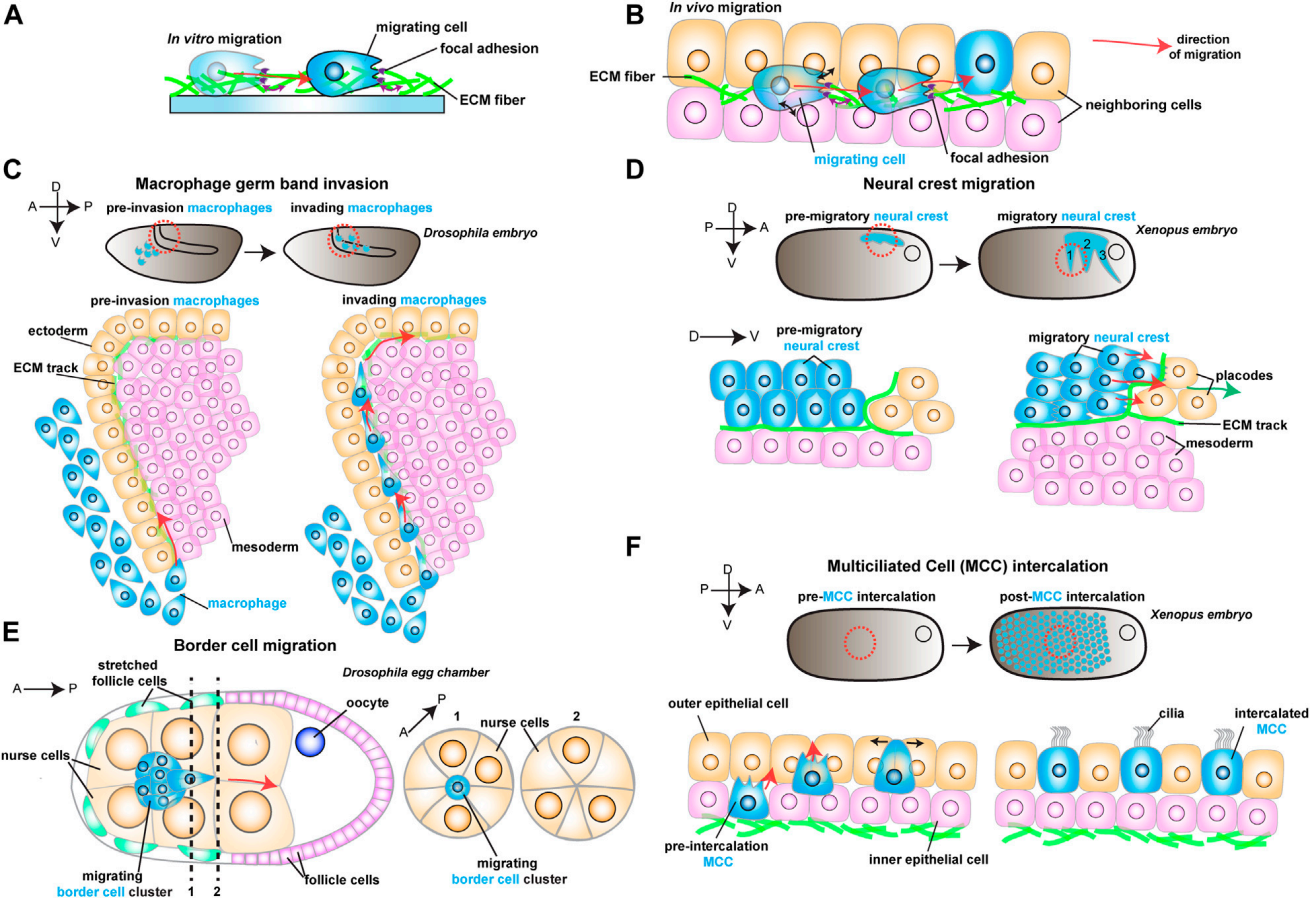
Schematic representation of different models to study *in vivo* cell migration. Red arrows show the direction of migrating cells. **(A)** Example of a cell (in blue) migrating *in vitro* on an ECM coated dish. As the cell moves, it reads mechanical cues from the ECM (purple arrows). **(B)** Example of a migrating cell (in blue) moving *in vivo* through complex environments composed of different cell types (in orange and pink) and extracellular matrix (ECM, in green). As the cell moves, it reads mechanical cues from the ECM (purple arrows) and the neighboring cells (black arrows). **(C)** *Drosophila* macrophage germ band invasion: (Top) Macrophages (in blue) migrate through the developing embryo and invade the germ band. (Bottom) Macrophages (in blue) invade the germ band by crawling through an ECM track laid between the ectoderm (in orange) and the mesoderm (in pink). **(D)** *Xenopus* Neural crest migration: (Top) Neural crest (NC) migrates as streams (in blue) along the embryo. (Bottom) NC cells (in blue) undergo epithelial to mesenchymal transition and chase after the placodes (in orange). The NC crawls on an ECM track (in green), which is laid on top of the mesoderm (in pink) and the placodes (in orange). **(E)** *Drosophila* Border cell migration: the border cell cluster (in blue) migrates through the middle of the egg chamber towards the posterior end, squeezing through the neighboring nurse cells (in orange). **(F)** *Xenopus* Multiciliated Cell intercalation: (Top) Multiciliated cells migrate to form the embryonic epidermis. (Bottom) Multiciliated cells (MCC) move from the inner epithelial layer (in pink) towards the outer epithelial layer (in orange), where they integrate the tissue by pushing the neighboring cells aside (black arrows).

The formation of organs during morphogenesis is an intricate process that relies on cells assembling into tissues, forming orderly units with defined shape and function. The mechanisms generating such complex collectives have long intrigued biologists. It is now clear that cell migration is essential for establishing and maintaining these diverse cellular architectures (Yamada and Sixt 2019). In its most simplified view, cell migration is initiated by the polarization of an individual cell (or group of cells) along a specific axis, propelled by actomyosin contraction and traction-generating actin-based protrusions that engage with the substrate, and directed by a gradient of biochemical cues (Figure 1A) (SenGupta, Parent, and Bear 2021). Recently, however, the importance of physical cues in cell migration has become apparent; the substrate’s physical properties in which cells migrate, such as ECM deformability (stiffness) and topography, play vital roles in cell migration (Charras and Sahai 2014; Helvert et al., 2018; Valet, Siggia, and Brivanlou 2022). Similarly, the native environment *in vivo* also exposes migrating cells to diverse mechanical stimuli. However, such environments are much more complex than their *in vitro* counterparts, as tissues are composed of different cell types and multiple ECM components that interact with the migrating cell (Figures 1A,B). Thus, our understanding of how cells sense and respond to the mechanical properties of their microenvironments *in vivo* is only starting to be defined. In this mini-review, we explore recent discoveries in different models of *in vivo* cell migration through confined environments. We then identify some common features illustrating how migratory behaviors depend on the physical interactions between migrating cells and their surroundings. Defining how such mechanical interplay is regulated will have major implications for understanding how migration shapes fundamental developmental processes, regeneration and cancer.

Cells moving through tissues are physically confined by their neighbors and components of the ECM (Figure 1B). In such crowded microenvironments, migrating cells must squeeze and push through as they move while simultaneously being exposed to various physical cues. We divide these signals into two broad categories: 1) spatial cues, which include the degree of physical constraint cells are exposed to (confinement), the specific features of the environment, such as the available space between neighboring cells (geometry) or how they are connected (topography), and 2) mechanical cues, which rely on the material properties of the substrate such as substrate stiffness to guide cell migration (durotaxis). In the first part of this mini-review, we describe examples of these types of physical information and how they guide cell migration. The second part describes how migrating cells respond to the microenvironment by changing their own mechanical properties. Finally, we provide a unifying perspective on the interplay between the behavior of migratory cells and the physical properties of the tissue through which cells migrate *in vivo*.

## The physical information of the 3D microenvironment

### Cellular confinement, geometry and topography as spatial cues

Cell migration through tissue environments proceeds through highly confining spaces. Confined 3D microenvironments present migrating cells with heterogeneous geometries from tight to broader spaces between cells or ECM pores (Figure 1B). While the development of microfabrication techniques has helped understand the impact of confinement *in vitro* (Reversat et al., 2020), the importance of mechanical confinement *in vivo* is still elusive.

**FIGURE 2:**
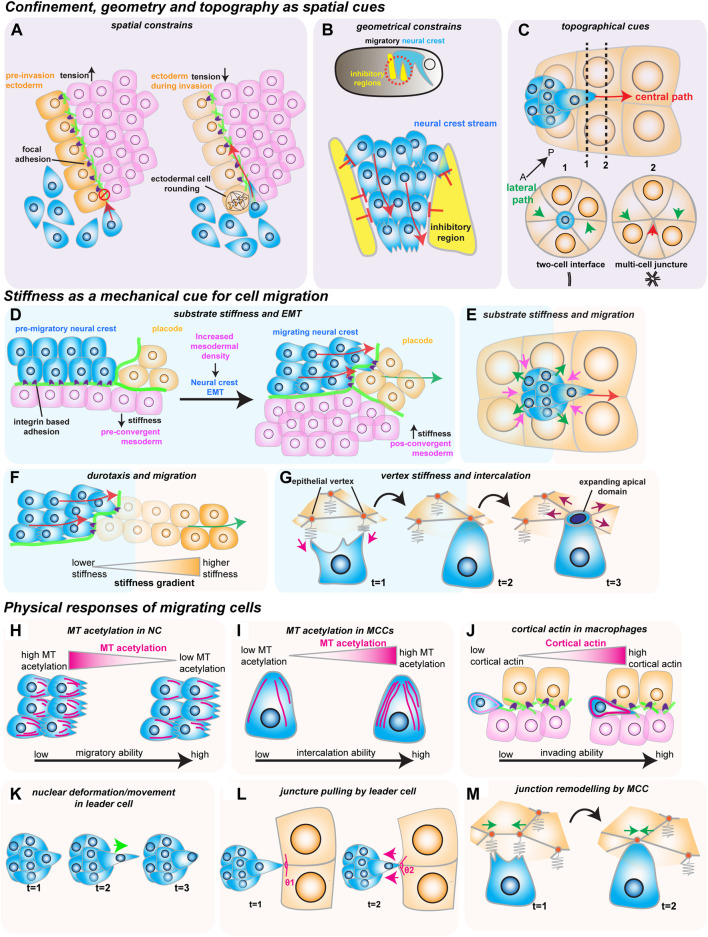
How mechanical cues impact cell migration *in vivo*. The red arrows show the direction of migrating cells. **(A–C)** Confinement, geometry and topography as spatial cues. **(A)** Spatial constraints block macrophage invasion (in blue) by controlling cells’ ability to crawl through the ectoderm (in orange) and the mesoderm (in magenta). Decreasing ectodermal tension is paired with ectodermal cell rounding to promote macrophage invasion. Ectodermal cell rounding removes focal adhesions (in purple) that act as an impediment to cell movement. **(B)** Geometric constraints control stream formation in the neural crest (in blue). Inhibitory signals (in yellow) regulate where NC can move, stopping NC cell dispersion and promoting collective cell migration. **(C)** Topographic cues determine border cell migration through the central path of the egg chamber (red arrow). The central path provides more space for cluster movement which is energetically favorable, as the multi-cell junctures are easier to unzip than to the lateral paths (green arrows), which are composed of tightly juxtaposed two-cell interfaces. **(D–G)** Stiffness as a mechanical cue for cell migration. **(D)** Increase in mesoderm stiffness induces NC EMT and migration. Convergent-extension movements increase cell density and stiffness of the mesoderm (in pink), which is sensed by the pre-migratory NC (in blue) through their integrin-based adhesions (in purple). **(E)** Nurse cell (in orange) stiffness impacts the migration of the border cell cluster (in blue). The compressive forces from the nurse cells (magenta arrows) are counteracted by the border cells (green arrows). **(F)** NC cells (in blue) interact with the placodes (in orange), which causes the placodes to retreat (green arrow), generating a stiffness gradient that directs cell migration. **(G)** Intercalating MCCs (in blue) pull on the vertices of the neighboring goblet epithelial cells (inorange) to sense vertex stiffness (magenta arrows). The stiffer multicellular vertices act as ideal entry points into the tissue as the increased total line tension favors the opening of the MCCs apical domain (purple arrows). H-M) Physical responses of migrating cells. **(H)** Microtubule (MT) deacetylation decreases NC stiffness to promote NC migration (acetylated MTs in magenta). **(I)** MT hyperacetylation promotes MCC intercalation, possibly by increasing cell stiffness (acetylated MTs in magenta). **(J)** Invading macrophages generate a protective cortical actin shell that shields the nucleus from compression. **(K)** In the migrating border cell cluster, the leader cell protrusions are stabilized by the active movement of the nucleus to the base of the protrusion (green arrow). **(L)** Border cells pull on the neighboring junctures of nurse cells to sense the environment (magenta arrows). **(M)** Intercalating MCCs remodel the neighboring goblet cell junctions (green arrows), promoting MCC intercalation.

There is no more intuitive example of confined migration than immune cells extravasating through vessels or moving through crowded tissues during immune surveillance. An emerging model of how cellular confinement controls immune cell migration *in vivo* is the macrophage invasion of the *Drosophila* germ band (Siekhaus et al., 2010). During *Drosophila* embryonic development, migrating macrophages distribute themselves across the embryo to ensure immune protection. A subset of the migrating macrophages invades the germ band by squeezing through the tightly juxtaposed ectoderm and mesoderm, with the invading macrophages extending protrusions and crawling along an ECM track (Figure 1C) (Ratheesh et al., 2018). Thus, migration through such cell-dense tissue could depend on the confinement and the mechanical features of the environment, concepts that remain poorly understood *in vivo*. Recent studies have identified two complementary mechanisms that promote macrophage migration. First, the ectodermal tissue tension is reduced by a decrease in myosin II contractility, which is triggered by the soluble factors released from the amnioserosal tissue neighboring the germ band (Figure 2A) (Ratheesh et al., 2018). This decrease in tissue tension facilitates macrophage invasion, as it enhances the ability of the ectodermal epithelium to deform in response to the invading macrophages. The second mechanism relies on local shape changes of the ectodermal cells at the entrance to the germ band (Akhmanova et al., 2022). As such, ectodermal cells act as gatekeepers to macrophage invasion, and when epithelial cells round up to divide, they form the entry points for macrophage invasion (Figure 2A). Consequently, inhibition of ectoderm cell division greatly blocks macrophage entry into the germ band. However, even in conditions where cell division is largely impaired, the few remaining macrophages still invade the germ band next to dividing or rounding cells, showing that ectoderm cell division is a decisive event for macrophage invasion. Notably, inducing ectoderm cell rounding is itself not sufficient to promote macrophage invasion. Rather, ectoderm cell rounding during division disassembles the focal adhesions (FAs) maintained by the ectodermal cells with the underlying ECM. These FAs impede macrophage entry by blocking the movement of the macrophages’ nucleus through the adhesion foci. Similarly, reducing FA components specifically in the ectoderm is sufficient to allow macrophages to invade the germ band, even in the absence of mitotic cell rounding.

While confinement can direct cell movement by controlling the amount of available space, cell migration can also be regulated by dictating where cells can effectively move through geometric constraints. The importance of geometric constraints is nicely illustrated in the migrating cephalic neural crest (NC) progenitors in the *Xenopus* embryo. This collectively migratory population moves along the ventral side of the embryo as it chases the neighboring placodes in well-defined streams (Figure 1D) (Theveneau et al., 2013). These streams rely on constrainment imposed by the surrounding tissues (Figure 2B) (Szabó et al., 2016). Although the constrainment in itself is not required for cell motility, it ensures directional collective cell migration, relying on the secretion of repellant signals such as semaphorins into the ECM by the neighboring cells. Semaphorins restrict the movement of NC cells by blocking the NC cells’ ability to make actin protrusions that provide traction, which avoids NC cell dispersion, keeping cells in the ideal path and ensuring efficient directional migration (Bajanca et al., 2019). Moreover, recent work has defined that the mechanosensitive ion channel Piezo1 in the NC cooperates with surrounding semaphorins, supporting the notion that mechanical cues can control directed cell migration (Canales Coutiño and Mayor, 2021). In this mechanism, Piezo1 is required to partially inhibit actin regulator Rac1 activity in the migrating NCs, which is reinforced by semaphorin signaling from the neighboring tissues. Together, Piezo1 and Semaphorins control protrusion dynamics to optimal levels, avoiding cell dispersion and sustaining directional migration.

While the geometrical features of the neighboring tissues can act as restricting cues, the microenvironment can also possess topographical features that serve as guidance signals for cell locomotion. A remarkably instructive system to tackle how topography regulates cell movement is border cell migration during *Drosophila* oogenesis. During ovarian development, the border cells form a cluster of six to ten epithelial cells that migrate towards the oocyte (Figure 1E) (Montell, Yoon, and Starz-Gaiano 2012). During migration, the border cell cluster moves through a highly constraining environment as the cluster squeezes through the surrounding nurse cells, which form the substrate on which cells migrate. Interestingly, while there are several paths border cells can migrate through, the cluster consistently moves through the center of the egg chamber as it advances towards the source of several chemotactic signals (Stuelten, Parent, and Montell 2018). While such signals are essential for the anterior-posterior (AP) movement, chemical cues do not explain why the border cell cluster consistently selects the central track. Recent work has tackled this question by reconstructing egg chambers in 3D and describing all possible paths for border cells inside the egg chamber (Dai et al., 2020). This detailed analysis determined that the central path is unique because it is where contacts (or junctures) between three or more nurse cells are enriched. This particular multiple-cell configuration is more spacious than the optional side paths, which are constituted by tightly juxtaposed two-cell interfaces (Figure 2C). Thus, the extra space provided by the central path originates a favorably energetic environment for the border cells to unzip the neighboring nurse cells (Figure 2C). The preferred central path illustrates how the steric constraints from the environment can direct cell migration.

### Stiffness as a mechanical cue for cell migration

As we have seen, geometrical and topographical features of the environment provide key spatial cues during *in vivo* cell migration. However, the rheological material properties, such as the substrate stiffness, can also serve as cues for cell movement. Indeed, migrating cells *in vitro* have long been known to respond to their environment’s stiffness (Charras and Sahai 2014; Janmey, Fletcher, and Reinhart-King 2020). Until recently, whether migrating cells *in vivo* also react to tissue stiffness had remained an open question. Intriguingly, NC cell migration *in vivo* depends on changes in the mechanical properties of the underlying mesoderm, which becomes stiffer prior to NC migration (Barriga et al., 2018). This increased substrate stiffness is sensed by the pre-migratory NC cells, causing them to undergo epithelial-to-mesenchymal transition (EMT), acquire motility and start migrating by extending actin protrusions that engage with the ECM substrate through focal adhesions (Figure 2D). The stiffening of the mesoderm driving this transition results from the increased cell density underneath the neural crest, as mesoderm cells undergo extensive convergent extension. Blocking convergent extension movements, or decreasing myosin II activity in the mesoderm, inhibits mesoderm stiffening, which blocks NC migration. Interestingly, the influence of substrate stiffness on cell migration is also observed in other model systems—border cell migration also depends on the stiffness of the nurse cells that form the substrate (Aranjuez et al., 2016; Lamb et al., 2021). However, contrary to the NC cells, increasing the myosin II activity in the nurse cells, and consequently their stiffness, blocks border cell migration (Figure 2E). These results suggest that substrate stiffness can act as a key regulator in cell migration and that changes in tissue stiffness elicit different responses from migrating cells that are context dependent.

As we have described, changes in the substrate stiffness control cells’ ability to migrate *in vivo*. Indeed, it has long been described that cells *in vitro* migrate along gradients of increasing substrate stiffness, in a process coined as durotaxis (Lo et al., 2000). While durotaxis has been well characterized *in vitro*, and substrate stiffness gradients *in vivo* have also been reported, whether cells migrate across stiffness gradients *in vivo* has long remained elusive (Shellard and Mayor 2021a). However, it has been recently shown that a durotactic gradient cooperates with chemotactic cues to direct NC migration (Shellard and Mayor, 2021b). As we have seen, NC cells migrate as a cluster along the dorsal-ventral axis as they chase the chemotactic signals secreted by the placodes, and move on ECM tracks laid out by the neighboring placodes and mesoderm (Figure 2D). Once the NC cluster reaches the placode, the two tissues establish repulsive interactions mediated by N-cadherin contacts. This causes the placodes to move away from the NC cells, which continually chase the placodal cells (Theveneau et al., 2013). Yet, during this interaction, NC cells also generate a stiffness gradient (Figure 2F) (Shellard and Mayor, 2021b). As the NC interacts with the retreating placodes through N-cadherin contacts, they cause the placodal cells they contact to soften. This induces the local generation of a stiffness gradient across the placode, which NC cells then sense through integrin-based adhesions (Figure 2F). Similarly to the chemotactic cues, the NC cells then persistently chase the retreating region of higher substrate stiffness. Interestingly, impairing either chemotaxis or durotaxis is sufficient to block proper NC migration, and neither tactic mechanism can overrule the other. It is then the coordinated chemotactic activity of the placodes and the self-generated stiffness gradient that moves the NC along the dorsal-ventral axis in a continuous mechanism of chase-and-run (Shellard and Mayor, 2021b).

While chemical and physical cues cooperate to sustain cell migration, it is unclear whether this is a universal principle. In some cases, mechanical signals might direct cell movement in the absence of clear chemotactic cues. One such example is the addition of multiciliated cells (MCCs) progenitors to the *Xenopus* embryonic epidermis, where no prevalent chemotactic signal has been defined (Stubbs et al., 2006; Werner et al., 2011; Collins et al., 2021). To join the epidermis, hundreds of MCCs execute the multi-step process of radial intercalation (RI) (Figure 1F) (Sedzinski et al., 2016). During RI, MCCs first move from the inner into the outer layer of the epidermis (Figure 1F). Once the MCCs reach the outer epithelium, they move toward the epithelial vertices formed by the outer epithelial cells, which constitute the entry points into the tissue (Figure 2G). Finally, MCCs emerge into the tissue by pushing the neighboring cells apart as they expand their apical domains. It is known that MCC apical emergence is initially dominated by pushing forces exerted by the MCC’s actin cortex, while pulling forces exerted by the neighboring epithelial cells contribute to completing the process (Figure 2G) (Sedzinski et al., 2016; Sedzinski et al., 2017; Kulkarni et al., 2021). Modulating the rigidity of the adjacent goblet cells is sufficient to control the final size of the MCC’s apical domain (Sedzinski et al., 2016). Thus, apical emergence depends on the fine balance between the mechanical properties of the intercalating cell and its epithelial neighbors. While apical emergence is a mechanical process, it is unclear whether mechanical cues inform MCCs where to intercalate. Recent work has shown that MCCs actively read the stiffness of the neighboring goblet cells to determine where to integrate (Figure 2G) (Ventura et al., 2021). As MCCs move apically, they extend actin-based protrusions (filopodia) that pull on the epithelial vertices of overlying goblet cells. Interestingly, the epithelial vertices constitute key mechanical hotspots within epithelia, suggesting that intercalating cells could use protrusions to pull and probe the mechanical environment (Higashi and Miller 2017). In silico experiments help explain that the pulling exerted by the MCC can be effectively used to measure the local stiffness of the epithelial vertices, which then determines where the MCCs integrate within the tissue. Vertices with higher stiffness are preferred positions for MCC intercalation because the combined higher line tension from the neighboring goblet cells’ junctions enhances apical expansion (Ventura et al., 2021). Thus, intercalating MCCs sense the stiffness of the neighboring cells to determine the ideal positions for cell intercalation.

## The physical responses of migrating cells to their surroundings

We have until now discussed how the surrounding environment’s mechanical properties control cell migration. Conversely, migrating cells read such mechanical cues and react to them with their own physical responses. As we shall see, these responses are diverse and range from migrating cells dynamically adjusting their mechanical properties to cells actively remodeling their surrounding environment (Figures 2H–M).

To start with the first example, it is now known that migrating cells fine-tune their mechanical properties in response to changes in the stiffness of the environment. As described above, an increase in mesoderm stiffness is required to induce NC EMT and migration (Barriga et al., 2018). However, recent work has shown that pre-migratory NC cells respond to the increased substrate stiffness by changing their mechanical properties (Marchant et al., 2022). In this mechanism, NC cells activate the mechanosensitive Piezo1 channel, causing NC cells to reduce microtubule (MT) acetylation (Figure 2H). This ultimately changes the mechanical properties of the NC by decreasing NC cell stiffness, which is required for NC migration, as sustaining MT acetylation blocks NC migration. Conversely, inducing a hypoacetylated microtubule network in NCs is sufficient to rescue migration in a non-stiff mesoderm. Thus, NC migration depends not only on changes in the surrounding tissues but also on a dynamic balance between the mechanical properties of the migrating cells and their substrate. This mechanism might not be unique to the neural crest, and a similar mechanism could be at play in other migrating cells. MCC intercalation also depends on the properties of its MT cytoskeleton, and MT hyperacetylation promotes MCC intercalation (Figure 2I) (Collins et al., 2021). How hyperacetylation determines the mechanical properties of the MCC, and whether this is a response to mechanical constraints from the neighbors, has not been defined. It is likely, however, that hyperacetylation sustains cortical rigidity in the MCCs as they push through their neighbors. Altogether, it is possible that controlling the properties of the MT network could provide a novel general mechanism for regulating the mechanical properties of migrating cells. Other components of the migrating cells’ cytoskeleton also play a crucial role in how cells physically respond to the environment. It is now known that the activity of actin bundling protein Fascin in the border cells is required to regulate the myosin II activity of the substrate nurse cells, effectively decreasing their stiffness (Figure 2E) (Lamb et al., 2021). Thus, the migrating border cells can actively fine-tune the stiffness of their substrate *in vivo*, and a complex force balance between the border cells and the nurse cells regulates border cell migration. Similarly, as we have seen, NC cells are able to soften the neighboring placodal cells, allowing them to establish the durotactic gradient that drives them along the dorsal-ventral axis (Figure 2F) (Shellard and Mayor, 2021b). Altogether, it is now clear that migrating cells actively respond to their environment *in vivo* by regulating their intrinsic mechanical properties. Moreover, while the individual mechanical properties of the substrate and the migrating cells are essential, it is the interplay between migrating cells and their substrate (a concept known as mechanoreciprocity) which is key to cell migration through complex tissue environments (Helvert et al., 2018).

As we have seen, the tissue environments through which cells move are highly confined, and migrating cells have to actively deform as they squeeze through the tissue. In this process, cells are particularly challenged by their nucleus, which is the largest and stiffest organelle and often the limiting step when cells migrate in 3D environments (Renkawitz et al., 2019). This forces cells to use strategies to mechanically adapt to such challenges. During macrophage invasion of the germ band, macrophages prepare for the challenging migration through the restrictive environment by forming a protective actin cortex (Belyaeva et al., 2022). This protective shell is required for proper macrophage migration, and it shields the migrating macrophage nucleus from mechanical stress during confinement. Macrophages fail to invade the germ band when the protective actin shell is lost (Figure 2J). A similar challenge is also faced by the migrating border cells. The relatively “spacious” central path border cells take is still smaller than the leading border cell. This requires the leader cell to squeeze its nucleus through the small available spaces (Penfield and Montell, 2021). As a response, the nucleus of the leader border cell deforms as it squeezes through the central path (Figure 2K). During this deformation, however, the leader cell nucleus controls the dynamics of the guiding protrusions. The nuclear movement to the base of the protrusion prevents protrusion collapse and facilitates growth, possibly by counteracting the rearward forces at the protrusion neck. While it can either act as a mechanical encumbrance or support, the nucleus also acts as a key mechanotransducer. Recent work has shown that nuclear stretching caused by compression, imitating the confinement experienced by cells, can activate myosin II activity and trigger cell migration (Lomakin et al., 2020; Venturini et al., 2020). Altogether, the nucleus is an active player in cell migration in a confined environment, with nuclear deformation mediating many important responses during migration.

Migrating cells also use other strategies to interact with the environment mechanically. As we have seen, actin-based protrusions provide traction during cell locomotion (Figure 1A). However, recently it has been shown how such actin protrusions can act as sensory organs, which actively pull on the neighboring cells (Figures 2G, L). Such actin protrusions pull on the neighboring environment to sense the available space in a possible path, as in the *Drosophila* border cells, or to probe the mechanical properties of the surrounding cells, as in the *Xenopus* MCCs (Dai et al., 2020; Ventura et al., 2021). Another exciting aspect of this phenomenon is how migrating cells can use such actin-based extensions to exert changes in the neighboring environment to promote cell migration. Recent work has defined how during intercalation, MCCs actively form higher-order vertices by inducing the remodeling of the junctions of its neighboring epithelial cells (Ventura et al., 2021). MCCs exert this out of plane remodeling by clutching and pulling the vertices at the ends of a junction, driving junction collapse to form the preferred higher order vertices (Figure 2M). Thus, MCCs can exert forces on the neighboring cells to generate a local environment that favors cell intercalation.

## Conclusion

Cell migration is one of the most fascinating and fundamental biological processes orchestrating our body plan. Understanding the complexity of cell migration *in vivo* requires studying the cellular dynamics locally, right in the microenvironment in which cells naturally reside. How does a heterogeneous microenvironment instruct cell migration? How do migrating cells respond to biomechanical cues presented by the surrounding microenvironment? These questions emphasize the need to consider cell migration as an interplay, a reciprocal interaction between a migrating cell and its surroundings. These interactions are dynamic and evolve over time as both the migrating cells and the microenvironment adapt their mechanical properties to fulfill an overarching developmental program. Furthermore, migrating cells not only read and respond but can also actively remodel the surrounding microenvironment, allowing them to trigger morphodynamic rearrangements efficiently. Recent work has also defined a whole new set of migrating cues, such as cell guidance by electrical gradients and pressure (Barriga and Theveneau 2020; Lennon-Duménil and Moreau 2021). Although these fall beyond the focus of this mini-review, they are incredibly interesting examples of the diversity of physical cues used by migrating cells to navigate complex environments. Altogether, studies addressing the mechanical interplay between migrating cells and the diverse environments they migrate through *in vivo* will provide important key insights into organ development, tissue homeostasis and disease pathology.
